# Effect of coronavirus (COVID-19) pandemic on orthopedic trauma patients presenting in the emergency department of a maximum care hospital and level 1 trauma center

**DOI:** 10.1007/s00402-021-04234-y

**Published:** 2021-11-09

**Authors:** M. Frink, V. Ketter, N. Klama, T. Knauf, S. Betz, S. Ruchholtz, R. Aigner

**Affiliations:** 1 Center for Orthopaedics and Trauma Surgery, University Hospital Giessen and Marburg GmbH, Location Marburg, Baldingerstraße, 35043 Marburg, Germany; 2 Center of Emergency Medicine, Philipps-University Marburg, Marburg, Germany

**Keywords:** Coronavirus, COVID-19, Emergency department, Orthopedic trauma

## Abstract

**Introduction:**

While overcrowding of emergency departments was often reported in the recent years, during the early phase of the pandemic, a reduction in patient numbers was seen. The aim of the current study was to describe the orthopedic trauma patient cohort presenting to the emergency department (ED) during the early pandemic period as compared to the cohort from the analogue time period 2019.

**Materials and methods:**

A single-center case–control study was performed. All the consecutive orthopedic trauma patients > 12 years presenting to the ED were included. Patients in the same time period in 2019 served as the control group.

**Results:**

Compared to 2019, in 2020, 33% less patients presented in the emergency department. Patients treated in 2020 were significantly older, significantly more often brought to ED by emergency medical services and significantly more often admitted. The number of fractures and diagnoses requiring surgical treatment decreased only slightly and the proportion of these patients among all the patients was significantly higher during the pandemic than in the control period. Furthermore, a higher percentage of polytrauma patients could be found in 2020 as well. Analysis of Manchester Triage System showed significantly less not urgent patients in 2020.

**Conclusion:**

The present study shows a significant decline in the number of patients treated in the ED during the pandemic period but at the same time almost identical numbers of patients with fractures or diagnoses requiring surgical treatment. In the context of an overall decline in patient numbers, a stronger concentration on level 1 trauma centers seems to be evident during the pandemic.

## Background

The current coronavirus pandemic represents a challenge for public health institutions. Therefore, unprecedented public and private restrictions were implemented in Germany.

In the course of this, elective diagnostic and therapeutic measures were restricted to keep existing resources available to patients with COVID-19 [1, 2].

The emergency department (ED) provides central medical help for private emergency attendance as well as emergency service admissions. Over the past decades, not only medical care got centralized, but an increase in emergency patients also occurred. Due to this development, frequently overcrowded EDs became a problem [3]. Factors contributing to ED overcrowding are widely discussed. Among other influencing variables, the increasing number of non-emergency patients attending the ED could be a reason. Access problems to general practitioners and medical specialists could encourage this way of seeking medical help. Internationally, overcrowding is considered as a serious issue to the public health system [4].

During the ongoing pandemic, the complete opposite could be observed. The number of self-referred attendance in the ED found to be reduced. The reasons for this are inconclusive. It could be either due to the decreased incidence of accidents or the increased concerns about getting infected by other ED patients.

The aim of this study was to characterize all patients presenting to our ED due to musculoskeletal problems during the pandemic period as well as comparing them to the patient characteristics in the same time period in 2019.

## Methods

This study was designed as a monocentric retrospective case–control-study at the interdisciplinary central ED of the University Hospital Giessen and Marburg GmbH, location Marburg. All patients over 12 years of age attending the ED due to musculoskeletal problems were included.

The University Hospital Giessen and Marburg GmbH, location Marburg represents a maximum care hospital. The center for orthopedics and trauma surgery is approved by the german association of trauma surgery (‘Deutsche Gesellschaft für Unfallchirurgie’ (DGU)) as a supraregional trauma center.

The period of observation spanned from 16.03.2020 to 10.05.2020. This time period represents the duration from the beginning of the lockdown until the first restrictions were suspended. The control period covered the same period in 2019.

Following parameters were collected and documented:

- Number of patients as well as gender and age
- Day of presentation, time of presentation
- Triage according to the Manchester Triage System
- Number of patients insured by the employer’s liability insurance association
- Duration of symptoms
- Fracture/type of fracture
- Inpatient admission
- Assigned by emergency service
- Mechanism of the accident
- Polytrauma and Injury Severity Score (ISS)

In addition, meteorological data, as well as public and school holidays, were identified in both observed periods.

### Statistics

Data were analyzed using IBM SPSS Statistics 24 (Statistical Package for the Social Sciences, IBM Corporation, Armonk, NY, USA). First, a descriptive statistic including the mean values and the standard deviation was carried out. We tested normality using the Kolmogorov Smirnov Test. In the case of a normal distribution, means were compared by *t* test. Otherwise, the comparison was carried out by the Mann–Whitney *U* test. A *p* ≤ 0.05 was defined as the significance level.

## Results

**Fig. 1:**
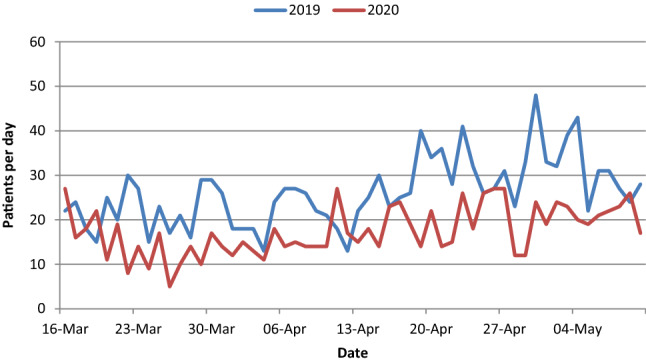
Comparison of the daily number of ED attendances 2019 and 2020

2441 patients with musculoskeletal problems presented to the ED of the (blinded) during the observed periods. While in 2019 1462 patients received treatment, in 2020 only 979 Patients attended this ED. This corresponds to a decline of 33%, representing a significant difference in the daily attendance number per day (see Fig. 1 and Table 1).

**Table 1:** Comparison of patient data 2019 versus 2020

	2019	2020	*p* value
Pat. per day	26,11	17,48	< 0.001
Gender (female/male)	671/791	475/504	0.203
Age	45.13 ± 23.61	50.53 ± 23.85	< 0.001
Time of presentation			0.104
8–16	751	460	
16–24	556	408	
0–8	155	111	
Weekend (yes/no)	433/1029	300/679	0.588
Transfer with Ambulance service (yes/no)	514/948	414/565	< 0.001
Duration of symptoms			0.387
< 24 h	887	607	
24–48 h	69	51	
48 h–7 days	159	86	
> 7 days	132	90	
Back pain as main symptom (yes/no)	124/1338	54/925	0.006
Inpatient admission (yes/no)	354/1108	299/680	0.001
Injury requiring surgical treatment (yes/no)	232/1230	214/765	< 0.001
Fracture (yes/no)	353/1109	323/656	< 0.001
Joint dislocation (yes/no)	53/1409	31/948	0.312
Work related injury (yes/no)	153/1309	37/942	< 0.001
Self harming (yes/no)	12/1450	13/966	0.223
Polytrauma (yes/no)	19/1443	25/954	0.022
Domestic violence (yes/no)	8/1454	8/971	0.418

The demographic parameters are shown in Table 1. The data shows a significant increase in the average age in patients in 2020. Concerning the time of presentation as well as the patients’ frequency of presentation at the weekend no differences could be found. Likewise, regarding the duration of symptoms before ED attendance no differences were observed, neither in self-referred patients nor in patients presented by emergency service.

However, there was a difference found in the ratio of patients presented by the emergency service. In 2020 a significant increased ratio of patients arrived at the ED by ambulance as compared to the reference year.

Based on the diagnosis data, a significant reduction of presentations due to back pain in the pandemic period could be found (*p* = 0.006). The ratio of inpatient admissions increased significantly in 2020 (*p* = 0.001). Furthermore, a significantly higher proportion of the patients presenting with a fracture to the ED was detected as compared to 2019. Work-related accidents in contrast were significantly less frequently treated in 2020.

Regarding the frequencies of specific accident mechanisms, such as self-harm or domestic violence, there could not be found year-on-year differences.

The different types of fractures are shown in Tables 2 and 3. There was no difference regarding the distribution of the presented fractures.

**Table 2:** Locations of fracture

	2019	2020
Femur	29	36
Humerus	26	20
Radius	58	66
Lower limb	13	13
Pelvis	11	13
Spine	43	29
Ankle	23	17
Foot	24	19
Ribs	22	24
Hand	47	50
Other	62	37

**Fig. 2:**
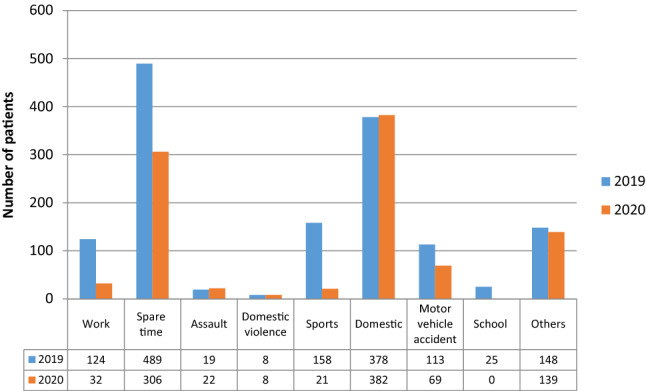
Cause of accidents compared between 2019 and 2020

The accident mechanisms leading to ED attendance are shown in Fig. 2. A decreasing number of traffic-, sports- and work-related accidents in the period of the pandemic were observed. On the other site, a significantly increased proportion of polytraumatized patients were treated in 2020 (*p* = 0.022) with a comparable injury severity demonstrated by the injury severity score (2019: 16.26 ± 7.32; 2020: 19.44 ± 9.39; *p* = 0.341).

**Table 3:** Geriatric fractures (age 65 and older)

	2019	2020
Proximal femoral fracture	22	29
Proximal humeral fracture	16	11
Spine fracture	34	28
Pelvic fracture	10	12
Periprosthetic fracture	2	6
Distal radial fracture	18	20
Total	102	106

Data regarding triage were available for 965 patients in 2020. Of those, 46 were categorized as red, 41 as orange, 387 as yellow, 451 as green and 37 as blue. In 2019, 1349 patients with triage data were collected. Thereby 41 were categorized as red, 42 as orange, 484 as yellow, 752 as green and 30 as blue. This corresponds to a significant difference in both observation periods (*p* < 0.001).

Regarding the meteorological parameters in 2020 the sun shone significantly longer per day (2019: 6.04 h ± 4.44 h; 2020: 8.98 h ± 4.11 h, *p* < 0.001). In 2019, a significantly increased amount of rain per day in mm could be found (2019: 1.05 ± 2.28; 2020: 0.68 ± 2.34; *p* = 0.031). Concerning the average temperature, no difference was found between both periods observed.

## Discussion

This study showed a decrease in the patients’ number treated due to musculoskeletal problems in the ED during the pandemic in 2020 compared to the same period in 2019. However, the number of fractures and diagnoses requiring surgical treatment decreased only slightly and the proportion of these patients among all patients was significantly higher during the pandemic than in the control period. Furthermore a higher percentage of polytrauma patients could be found in 2020 as well.

Overall there was a one third decrease of patients treated at our ED due to musculoskeletal problems, which was mainly due to a decreasing number of patients not requiring acute inpatient treatment. This decrease of emergency patients is in line with other studies from other level one trauma centers in Germany which reported a similar reduction in emergency patients [5–7]. A similar extent of the reduction in presentations was also found in a study from Spain. It reports a reduction of 37% in orthopedic and trauma surgery patients at the ED [2]. During the period of the corona pandemic, the ED plays a pivotal role—not only for the treatment of patients suffering from COVID-19 with and without symptoms, but also for non-infectious patients [8]. This close interaction as well as the resulting fear of getting infected should be discussed as a possible reasons for the decline in the patient numbers. However the patient number reduction could also be caused by the citizens’ conscientiousness to follow the imposed rules of “social distancing” and “stay at home”.

This study showed an only slight decrease in the number of patients who have presented themselves based on surgery requiring problems and fractures. Furthermore, only a minor reduction in inpatient admissions could be found. The rather small reduction in inpatient admissions from the emergency department compared with the overall patient reduction is consistent with a comparable study by Wähnert et al. [6]. This decline may be explained by the lower activity of citizens at the time of the restrictions associated with the Corona pandemic. However in this study the proportion of patients suffering from fractures or diagnoses requiring surgery was increased. This is in line with the results of a study by Kreis et al. which reported an increased incidence proportion of emergency operations during the shutdown [5].

Patients suffering from less urgent, ambulant treatable conditions however attended the ED less often during the pandemic in this sample. In accordance with these findings Maleitzke et al. showed a decrease in the incidence proportions of patients presenting to the ED with non-traumatic orthopedic symptoms during the shutdown period. More specifically a decrease of patients presenting with unspecific pain was found [7]. In the present study in comparison to 2019, a significant decline in presentations due to back pain could be found in 2020. In addition, regarding the triage categories according to the Manchester Triage System, a smaller proportion of less acute patients were treated in the ED in 2020. It seems probable that a higher individual barrier for presentation to the central ED of a maximum care hospital was present in patients with nonurgent conditions.

A decline in certain injury mechanisms throughout the pandemic could be an additional reason for this reduction. According to estimates by the Federal Institute for Occupational Safety and Health, the cause of accident injuries is to be found in almost 64% of all accidents in the areas of leisure, work or school [9] which were influenced by the imposed restrictions. Many people did not routinely exercise their profession during this period. In addition, other activities commonly associated with a certain risk of injury, such as recreational sports, were also largely restricted. The legal restrictions can certainly be assumed as the main reason for the significant reduction in the number of patients treated in ED, which were insured by the employer’s liability insurance association. In addition, the lack of school accidents due to the nationwide closure of schools further contributes to the reduction of the number of these patients. In the present study, a decreasing number of traffic-, sports- and work-related accidents in the period of the pandemic were observed which is also in accordance with previous studies [5, 7].

A slight increase in the total number of polytrauma patients was found. This represents a significant increase in the proportion of polytrauma patients among all patients treated in 2020 compared with 2019. This increase is in line with another analysis from a German level one trauma center [6]. A further retrospective analysis from a German level one trauma center found no substantial difference in the total number of seriously injured patients when compared to the years 2018 and 2019 [10]. In line with the results of the current study the mean ISS showed no significant differences in previous studies [6, 10].

Overall, in accordance with other studies from other level one trauma centers in Germany, there is a reduction in the total number of patients with a comparable number of patients with polytrauma and fractures. In contrast, a study by Kuhlen et al., which analyzed the data of the optional external quality program IQM found a decrease of femoral neck fractures of nearly 24% and of trochanteric femur fractures of nearly 19% [11]. This is also supported by the results of Schoeneberg et al. They conducted an online survey of all registered German Trauma Centers and Geriatric Trauma Centers [12]. These results do not only represent results from maximum care hospitals and Level 1 hospitals, but from all levels of health care in Germany. Therefore, it seems likely that the reduction in diagnoses requiring treatment primarily affected hospitals from the lower care levels.

At the beginning of the pandemic, there were warnings of a possible increase in domestic violence and suicides due to the circumstances of the restrictions imposed. However in the present study such an increase could not be observed. In contrast to the results of our study, which was conducted in a relatively rural area, data from Berlin showed a significant increase in the rate of injuries resulting from domestic violence [7].

The present study suffers from limitations. First, the retrospective study design needs to be critically discussed. Furthermore, the results of the study only represent the reality of one university medical center providing maximum care in a rural region. Further studies including hospitals of all levels of care in various regions are required to confirm the results mentioned above. However, this study is based on a detailed analysis of consecutive patients at the time of the corona pandemic, as well as the corresponding period in 2019. Thus, a considerable number of patients were included allowing statistical comparisons and conclusions to be drawn regarding the impact of the pandemic.

## Conclusion

The present study shows a significant decline in the number of patients treated in the ED during the pandemic period but at the same time almost identical numbers of patients with fractures or diagnoses requiring surgical treatment. In the context of an overall decline in patient numbers, a stronger concentration on level 1 trauma centers seems to be evident during the pandemic.
